# Machine learning‐assisted optimizing dietary intervention against dementia risk

**DOI:** 10.1002/alz70861_108234

**Published:** 2025-12-23

**Authors:** Jin‐tai Yu

**Affiliations:** ^1^ National Center for Neurological Disorders, Shanghai, China; Huashan hospital, Fudan University, Shanghai China

## Abstract

**Background:**

Dietary factors play a key role in the development of dementia, but the optimal dietary pattern for its primary prevention has yet to be determined.

**Method:**

Utilizing a three‐step approach, we devised a Machine learning‐assisted Optimizing Dietary intERvention against demeNtia risk (MODERN) dietary pattern using data from 185,012 UK Biobank participants, among whom 1,987 developed incident all‐cause dementia (ACD) during the 10‐year follow‐up.

**Result:**

We first applied a food‐wide association analysis and identified 25 out of 34 food groups linearly or non‐linearly associated with incident ACD. We then adopted a machine learning‐based approach to rank their importance in predicting future ACD risk, and eight food groups (including poultry, potatoes, eggs, citrus fruits, sweetened beverages, olive oil, berries, and green leafy vegetables) entered the new dietary pattern. Rating the intake levels of the selected food groups, we established a MODERN diet score (range: 0‐7) and externally validated its association with ACD risk in three independent cohorts. The MODERN diet score demonstrated a significant association with dementia‐related outcomes (Hazard ratio [HR]_T3vsT1_: 0.64; 95% confidence interval [CI]: 0.43‐0.93; HR per 20% increment: 0.74), which was stronger than the *a priori*‐defined Mediterranean‐DASH Intervention for Neurodegenerative Delay diet score (HR_T3vsT1_: 0.75; 95%CI: 0.61‐0.92; HR per 20% increment: 0.78). Furthermore, an outcome‐wide association analysis spanning 53 health‐related outcomes revealed the wide‐range protective associations of the MODERN diet score, especially with mental and behavioral disorders. We also identified potential pathways through brain imaging, inflammation, metabolomics, and proteomics analyses.

**Conclusion:**

Overall, the devised MODERN diet may serve as a potential dietary approach for the primary prevention of dementia.

